# Professional barriers and facilitators to using stratified care approaches for managing non-specific low back pain: a qualitative study with Canadian physiotherapists and chiropractors

**DOI:** 10.1186/s12998-019-0286-3

**Published:** 2019-12-13

**Authors:** Fadi M. AL Zoubi, Simon D. French, Andrea M. Patey, Nancy E. Mayo, André E. Bussières

**Affiliations:** 1 School of Physical and Occupational Therapy, Faculty of Medicine, McGill University, 3630 Promenade Sir-William-Osler, H3G 1Y5, Montreal, QC Canada; 2 Centre de recherche interdisciplinaire en réadaptation (CRIR), Montréal, QC Canada; 3 Department of Chiropractic, Macquarie University, Sydney, NSW Australia; 4 School of Rehabilitation Therapy, Queen’s University, Kingston, ON Canada; 5 Centre for Implementation Research, Ottawa Hospital Research Institute, Ottawa, ON Canada; 6 Département chiropratique, Université du Québec à Trois-Rivières, Trois-Rivières, QC Canada

**Keywords:** Knowledge translation, Interviews, Stratified care approach, Low Back Pain, Chiropractic, Physiotherapy, Evidence-based practice, Theoretical domains framework

## Abstract

**Background:**

Recent clinical practice guidelines for the management of non-specific low back pain (LBP) recommend using stratified care approaches. To date, no study has assessed barriers and facilitators for health professionals in using stratified care approaches for managing non-specific LBP in the Canadian primary care setting. This study aimed to identify and contrast barriers and facilitators to using the stratified care approaches for non-specific LBP among Canadian physiotherapists and chiropractors.

**Methods:**

Individual telephone interviews, underpinned by the Theoretical Domains Framework (TDF), explored beliefs and attitudes about, and identified barriers and facilitators to the use of stratified care approaches for managing non-specific LBP in a purposive sample of 13 chiropractors and 14 physiotherapists between September 2015 and June 2016. Interviews were digitally recorded, transcribed verbatim and analysed by two independent assessors using directed content analysis.

**Results:**

Three and seven TDF domains were identified as likely relevant for physiotherapists and chiropractors, respectively. Shared key beliefs (and relevant domains of the TDF) for both physiotherapists and chiropractors included: lack of time, cost, and expertise (*Environmental Context and Resources*)*;* and consulting more experienced colleagues and chronic patients with important psychological overlay (*Social Influences*)*.* Unique key domains were identified among physiotherapists: incompatibility with achieving other objectives (*Goals*), and chiropractors: confidence in using stratified care approaches (*Beliefs about Capabilities*)*;* intention to use stratified care approaches (*Intentions*)*;* awareness and agreement with stratified care approaches (*Knowledge*)*;* assessment of readiness for change and intentional planning behaviour (*Behavioural Regulation*)*;* and improving the management of non-specific LBP patients and the uptake of evidence-based practice (*Beliefs about Consequences*).

**Conclusions:**

Several shared and unique barriers and facilitators to using the stratified care approaches for non-specific LBP among Canadian physiotherapists and chiropractors were identified. Findings may help inform the design of tailored theory-based knowledge translation interventions to increase the uptake of stratified care approaches in clinical practice.

## Background

Low back pain (LBP) is the leading cause of disability, affecting over 630 million people worldwide [1], and results in significant burden and high cost to society [2]. LBP is associated with a wide range of physical, psychological, and social influences on patients’ lives including functional disability, depression, work absence, poor productivity, poor quality of life, and increased healthcare utilization [3–8]. Out of 328 medical conditions, LBP was found to be the top contributor to global disability in the 2016 global burden of disease study [9]. In the U.S., the direct and indirect costs of LBP are estimated to exceed $100 billion per year [10, 11], while in Canada, the estimates of the healthcare cost of LBP range between $6 and $12 billion annually [12].

Non-specific LBP is a broad diagnostic term referring to pain that cannot be attributed to any specific cause or pathology [13]. Approximately 90% of all LBP cases presenting to primary care are non-specific in nature [14]. However, a cross-sectional study in Australia of six health disciplines including physiotherapists and doctors of chiropractic suggested that 93% of the clinicians did not view acute non-specific LBP as a single entity [15]. A majority (74%) of respondents thought it is possible to recognize subgroups among non-specific LBP patients.

In spite of significant research efforts to better understand the causes of non-specific LBP, identifying the exact source of “pain generators” remains challenging. It has been argued that in the absence of a clear diagnosis, appropriate treatment plans and care can be difficult to prescribe [16]. This has led to a large body of literature evaluating “one-size-fits-all” approach to treat patients in this category, despite their widely recognized heterogeneity [17]. Some argue that this “one-size-fits-all” approach can lead to diagnosis-treatment mismatch [18].

To improve the diagnosis-treatment process, various stratified care approaches, also known as classification systems, have been proposed to improve patient care and reduce levels of disability for non-specific LBP [19–23]. These stratified care approaches recommend different ways of assessing and treating non-specific LBP by attempting to classify patients into clinically relevant sub-groups who can then receive targeted interventions [24]. One of the first stratified care approaches was developed by Robin McKenzie in 1981 [25] where patients are sub-grouped based on three clinical presentations: postural syndrome, dysfunction and derangement. Since then many more stratified care approaches have emerged, with Billis et al. [19] identifying 39 stratified care approaches from nine countries, and a number of additional stratified care approaches have been proposed since [21–23]. Fairbank et al. (2011) [21] identified 28 stratified care approaches (16 diagnostic, 7 prognostic, and 5 treatment-based) specifically for the management of chronic non-specific LBP, while three clinical practice guidelines on the management of LBP suggested the use of the stratified care approaches to subgroup non-specific LBP patients [24, 26, 27]. However, no particular stratified care approach is suggested as being superior over others.

Despite the potential usefulness of stratified care approaches for managing non-specific LBP, many physiotherapists continue to be divided on the use of such approaches [28]. Some choose to continue managing non-specific LBP patients using interventions with limited or no evidence of effectiveness [29], even though patients fail to show any improvement after 3 months [30]. In one study in Quebec, about one third of physiotherapists indicated using the McKenzie subgrouping approach [29] and those physiotherapists were recent graduates or regularly attended postgraduate clinical education [29]. While the uptake of stratified care approaches in chiropractic practice is currently unknown, it is unlikely to be higher considering that stratified care approaches were mostly developed by physiotherapists.

Understanding the varying uptake of stratified care approaches in clinical practice could inform the design of more effective implementation strategies. Among the stratified care approaches recommended by clinical practice guidelines, some focus on diagnostic, prognostic, or treatment stratification. However, there is no cut evidence that one approach is superior over other. For instance, the STarT Back Screening Tool has a level Ia (high quality) evidence for its clinical- and cost-effectiveness [31]. STarT Back provides a systematic method of assessing the biopsychosocial prognostic factors of delayed recovery and matching certain intervention accordingly [32]. On the other hand, a recent meta-analysis [33] concluded that in patients with chronic LBP, McKenzie Method of Mechanical Diagnosis and Therapy is superior to other rehabilitation interventions; however this is dependent on the other kind of intervention being compared to. McKenzie Method was not superior to other rehabilitation interventions for acute LBP. In summary, none of the stratified care approached has shown superiority for all cases of LBP and their focus varies. Furthermore, clinicians’ exposure to any particular stratified care approaches during their training or after graduation is highly variable. For these reasons, we did not limit the behaviour of interest to any one approach. To date, no study has assessed individual barriers and facilitators to using stratified care approaches for managing non-specific LBP among Canadian physiotherapists and chiropractors. Our aim was to explore the determinants of using any of these approaches among physiotherapists and chiropractors. We used the theoretical domains framework (TDF) [34, 35] to explore clinicians’ beliefs and attitudes about, and barriers to, the use of stratified care approaches. The TDF was developed to assess factors likely to impede or enable the uptake of clinical practice guidelines and best practices, and to “simplify and integrate a plethora of behaviour change theories and make theory more accessible to, and usable by, other disciplines” (p. 2) [34]. The TDF has been used across various healthcare settings, health disciplines and clinical conditions to provide in-depth understanding of the underlying behavioural problem and to suggest interventions to increase the likelihood of successful implementation [36–42]. A three-step validation of this framework resulted in a revised version of the TDF which contains 14 domains [34].

### Objectives

This study aimed to identify the perceived barriers and facilitators to using the stratified care approaches for managing patients with non-specific LBP among physiotherapists and chiropractors in Canada.

## Methods

### Design

This was a qualitative study using semi-structured interviews among Canadian physiotherapists and chiropractors. The specified target behaviour was “Using stratified care approaches to tailor treatment for non-specific LBP patients among Canadian physiotherapists and chiropractors”.

### Ethics

All participants provided informed consent. Approval for this project was granted by McGill Institutional Review Board (IRB Study Number A09-B49-15B).

### Study population and eligibility criteria

Participants were recruited from the member lists of the Canadian Physiotherapy Association and the Canadian Chiropractic Association. To ensure that a variety of viewpoints were represented, respondents were approached across a wide spectrum of geographical areas, physical therapy and chiropractic school attended, type of practice, years in practice, and expertise (clinicians, clinical instructors, and faculty). Purposive and snowballing sampling were used to recruit clinicians. Invitations to participate were also sent through LinkedIn and Facebook. Eligible participants had to be licensed physiotherapists or chiropractors in full- or part-time practice and consulted patients with LBP. Providers seeing on average fewer than two new LBP patients per week were excluded.

As proposed by Francis et al. (2010) [43], 10 clinicians (for each health discipline) were interviewed for the initial analysis sample (with appropriate diversity sampling). Thereafter, three more interviews were conducted and when no additional themes emerged, this was identified as the point of data saturation. This stopping criterion was tested after each successive interview (i.e. 11, 12 and 13; then 12, 13 and 14, and so on) until there were three consecutive interviews without additional themes.

### Procedures

A sample of 70 practitioners (35 physiotherapists and 35 chiropractors) was invited by e-mail to take part in telephone interviews between September 2015 and June 2016. The first 15 respondents in each discipline (PT and DC) were followed up by email or telephone to discuss their participation. Those who agreed to participate were asked to complete a consent form. Interviews were conducted by two researchers (one investigator (FAZ)¸ a male with clinical training in physiotherapy and a female research assistant with graduate training, both familiar with the TDF and theoretical coding) for a duration of approximately 45 min. Field notes were taken during interviews. All interviews were digitally and transcribed verbatim by the same research assistant.

As an incentive to participate, names of respondents were entered into a draw shortly after the interviews to win one of two $100 gift certificates.

### Material

A semi-structured interview guide for clinician (PT and DC) interviews (see Additional file 1) was developed based on the revised TDF [34, 44] containing 14 domains: *Knowledge, Skills, Social/Professional Role and Identity, Beliefs about Capabilities, Optimism, Beliefs about Consequences, Reinforcement, Intentions, Goals, Memory, Attention and Decision Processes, Environmental Context and Resources, Social Influences, Emotions, and Behavioural Regulation.* The number of questions ranged from one to four for each of the 14 TDF domains for a total of 30 questions. Questions were informed by previously published work on the topic [45–50]. Face and content validity of the interview guide were initially assessed by co-authors, who have experience in knowledge translation (KT) and the use of the TDF (AEB, SDF, FAZ, and AMP).

### Data analysis

As this study is using TDF, directed content analysis was used where our coding was relevant to the theoretical domains as prescribed by Hsieh & Shannon [51]. One investigator (FAZ) and a research assistant, who are familiar with the TDF domains and theoretical coding, independently coded all the transcripts in light of the relation between the utterances and the specific behaviour. Disagreements were formally resolved at each step. Each utterance was first coded into the relevant theoretical domains from the TDF onto an Excel spreadsheet. Utterances were counted twice if a participant provided a response similar to that of another participant. Utterances unlikely to be relevant to the use of stratified care approaches were discarded. Three experts in knowledge translation (KT) and TDF (AEB, SDF, AMP) provided a critique of the analysis and reviewed 15% of randomly selected coding to ensure a robust and defensible coding of the data into beliefs and relevant domains.

We used similar analysis strategies as in prior related work [44, 52]. Briefly, utterances were then linked to specific beliefs. A specific belief is a statement that gives some details regarding the role of the domain in terms of influencing the behaviour [49]. Such statements convey a meaning/theme that is common across multiple utterances. Beliefs, coded as being similar or identical statements, were grouped together according to their likelihood to either increase (i.e., perceived to facilitate or help the use of stratified care approaches), decrease (i.e., perceived barriers to the use of stratified care approaches), or have no influence on the target behaviour. Two to three emerging, overarching themes were proposed for each domain. Specific beliefs and overarching themes were then reviewed for agreement.

Finally, relevant domains were identified based on the following three criteria and weighted equally to permit a decision regarding the relevance of the domains and whether they influenced the target behaviour: 1) presence of conflicting beliefs, 2) evidence of strong beliefs that were perceived to impact the behaviour, and 3) high frequency of specific beliefs [49, 51].

## Results

### Characteristics of participants

Telephone interviews were conducted with 14 physiotherapists from four Canadian provinces [Quebec (*n* = 2), Ontario (*n* = 7), Nova Scotia (*n* = 1), and Alberta (*n* = 4)], and 13 chiropractors from four Canadian provinces, including [Quebec (n = 2), Ontario (*n* = 8), Manitoba (n = 1), and Alberta (n = 2)]. The average age of participants was 39.3 years (SD ± 7.97), 41.8 years (SD ± 9.04) for physiotherapists and chiropractors respectively. The percentage of females among physiotherapists was 64.3% (9/14) while for chiropractors it was 15.4% (2/13). The average number of years in practice was 14.8 years (SD ± 8.9) and 14.8 years (SD ± 10.2) for physiotherapists and chiropractors respectively (Table 1).

**Table 1:** Professionals’ characteristics

Characteristics	Physical therapists (*N* = 14)	Chiropractors (*N* = 13)
Age	39.3 years (SD ± 7.97)	41.8 (SD ± 9.04)
Gender (n (%))		
Male	5 (35.7)	11 (84.6)
Female	9 (64.3)	2 (15.4)
Highest level of education		
Diploma Program		
Undergraduate Degree	8	10
Master Degree	5	3
PhD Degree		
Clinical experience (years)	14.8 (SD ± 8.9)	14.8 (SD ± 10.2)
Current employment status		
Full-time^a^	10	10
Part-time^b^	4	3
Practice type /workplace?		
Solo practice	3	2
Group practice	11	11
Clinical setting		
Private	7	7
Multidisciplinary health care center	4	6
Rehabilitation center	1	
Hospitals	2	
Socioeconomic status (SES) of managed patients (on average)		
Mostly high SES	4	7
Middle SES	9	5
Mostly low SES	1	1
Practice location		
Urban area	7	7
Suburban area	5	6
Rural area	2	
Average total number of patients (new and regular visit) per week	39	67
Average total number of LBP patients (new and regular visit) per week	13	34
Average number of NEW cases of LBP per week	3	2

^a^more than 16 hours in patient- contact hours; ^b^ less than 16 hours in patient- contact hours; *LBP* low back pain

### Key themes identified within relevant domains

Among physiotherapists, we identified 421 statements representing 32 specific beliefs and 18 themes. Three key domains were identified as relevant to changing the targeted behaviour: *Goals; Environmental Context and Resources; and Social Influences*. The other 10 domains were considered to have a lesser influence on changing the targeted behaviour. Table 2 presents the specific beliefs and relevant domains for physiotherapists. Findings of relevant domains together with illustrative quotes for physiotherapists are provided in Additional file 2.

**Table 2:** Thematic content analysis based on the TDF for physiotherapists

Domain	Questions (N)	Utterances (N)	Specific beliefs (N)	Specific beliefs (number of utterances)	Increase^a^ N (%)	Decrease^b^ N (%)	No Influence^c^ N (%)	Themes
Knowledge	4	56	4	I am aware of existing SCA (14)	56 (100)	0	0	Awareness of SCAs; Knowledge of evidence
				My understanding about the use of SCA is to classify patients into groups to provide effective treatment for each group. (14)				
				I agree with the recommended use of SCA for LBP patients. (14)				
				I know how to use SCA to target the management of non-specific LBP patients. (14)				
Belief about consequences	3	44	3	I believe the benefits of using SCA include empowering patients to self-manage, more accurate assessment, better matching of treatment, minimizing visits and costs, increasing self-efficacy, less passive treatment. (16)	44 (100)	0	0	Consequence of managing patients with/without SCAs
				I believe the disadvantages of not using SCA include slower recovery, lower patient satisfaction, less self-management and autonomy, longer treatment time, higher costs, poorer standard of care. (14)				
				Outcomes I expect to see are less pain, better function, faster recovery, adherence to protocols, self-management, higher satisfaction, faster return to work, fewer visits, less medication. (14)				
Belief about capabilities	1	14	1	I am confident in assessing NSLBP patients using SCA & determining the targeted treatments. (14)	14 (100)	0	0	Acceptance, capabilities
Behavioural Regulation	3	31	3	I do (13)/ don't (1) have strategies to monitor changes in patients’ health status	26 (84)	0	5 (16)	Assessing readiness for change; Intentional planning behaviour
				It would be helpful to have: more subjective and objective exams (2), team work (1), and awareness from other stakeholders (1).				
				I have a clear plan under what circumstances I will use SCA in my practice. (13)				
Skills	4	53	4	I have been trained to use SCA (14)	41 (77)	0	12 (23)	Clinical training; Clinician-Patient and clinician –clinician communication skill
				I have the necessary skills to use SCA (13)				
				Skills required to treat patients with high risk of disability are: ability to screen, good communication, psychosocial training, teamwork. (12)				
				Communication skills are extremely important for the management of LBP patients using SCA. (14)				
Intention	1	14	1	I will manage all (10)/ most (4) of the next 10 patients using SCA	14 (100)	0	0	Decision to manage patients using SCAs
Goals	1	16	1	The goal of managing NSLBP patients with SCA is (12)/ not (4) incompatible with achieving other objectives.	4 (25)	12 (75)	0	Incompatibility with achieving other objectives
Memory, Attention & Decision	2	28	2	Deciding if a patient should be managed using SCA is easy (11)/ not easy (1).	27 (96)	1 (4)	0	Ease of decision
				The rule of thumb I use to guide my decision making for the patient care is: the SCA itself (11), research and effectiveness (1), the mechanical component in the history (2), or patient compliance (2).				
Reinforcement	1	14	1	I would manage NSLBP most of the time using the SCA because rewards are greater and patients are satisfied. (14)	14 (100)	0	0	Better outcomes reinforce the use of SCAs
Environmental Context and Resources	3	52	5	Barriers to using SCA include lack of time, cost, other colleagues who do not use SCA, lack of expertise, patient preference, language, and unmotivated patients. (16)	34 (65)	17 (33)	1 (2)	Environmental resources
				No barriers to using SCA. (1)				
				Facilitators to using SCA include: need for fewer sessions, having private room, autonomy, team work, and support from management. (6)				
				No (13)/ some (1) onsite rehabilitation equipment is required.				
				There are resources available that help me manage patients using the SCA. (15)				
Social Influences	4	58	4	I would (9)/ not (4) consider consulting more experienced practitioners if I need help.	41 (71)	10 (17)	7 (12)	Influence of colleagues and researchers; psychological cases influence decision
				The views of other colleagues (9)/ researchers (7) influence my decision to manage patients using SCA.				
				Having an acute patient in apparent distress would (1)/ would not (13) influence my decision to manage such patients using the SCA.				
				Having a chronic patient with important psychological overlay would (5)/ would not (10) influence my decision to manage with SCA.				
Optimism	1	14	1	I am generally optimistic (13)/ not sure (1) regarding the added value of using SCA, in my daily practice.	13 (93)	0	1 (7)	Positive attitude
Social Professional identity	2	27	2	I consider using SCA to be part of my work as a physiotherapist. (13)	27 (100)	0	0	Professional role; Professional agreement
				I think it is appropriate that my role should include managing patients with non-specific LBP using the SCA. (14)				

^a^Statements perceived to increase use of stratified care approaches (facilitators). ^b^ Statements perceived to reduce use of stratified care approaches (barriers). ^c^ Statements perceived to neither increase/decrease the use of stratified care approaches. *SCA* stratified care approach, *LBP* low back pain.

Among chiropractors, we identified 411 statements representing 35 specific beliefs and 18 themes. Seven key domains were considered to have a greater influence on the targeted behaviour for chiropractors: *Environmental Context and Resources; Beliefs about Capabilities; Social Influences; Intentions; Knowledge; Behavioural Regulation; and Beliefs about Consequences*. The six other domains were considered to have a lesser influence on the targeted behaviour. Table 3 presents the specific beliefs and relevant domains for chiropractors. Findings of relevant domains together with illustrative quotes for chiropractors in Additional file 3.

**Table 3:** Thematic content analysis based on the TDF for chiropractors

TDF Domain	Questions (N)	Utterances (N)	Specific beliefs (N)	Specific beliefs (Number of utterances)	Increase^a^ N (%)	Decrease^b^ N (%)	No Influence^c^ N (%)	Themes
Knowledge	4	51	4	I am aware of existing SCA (13)	40 (78)	9 (18)	2 (4)	Awareness of SCAs; Knowledge of evidence
				My understanding about the use of SCA is: to classify patients into groups to provide effective treatment for each group (9), It can streamline different professionals' work (1), it has different types (1), and it is not understood for me (1).				
				I do (11)/ do not necessarily (2) agree with the recommended use of SCA for LBP patients.				
				I know (7)/ don't know (2)/ know but not necessarily (4) use SCA to target the management of non-specific LBP patients				
Belief about consequences	3	58	3	I believe the dis (7)/advantages (27) of using SCA include management of patients and evidence-based practice	51 (88)	7 (12)	0	Consequence of managing patients with/without SCAs
				I believe the disadvantages of not using SCA include poor management of patients and not evidence-based practice (12)				
				Expected outcomes: less pain, better function, faster recovery, higher satisfaction, and less medication (12)				
Belief about capabilities	1	17	2	I am confident (11)/not confident (4) in assessing NSLBP patients using SCA & determining the targeted treatments	11 (65)	6 (35)	0	Acceptance, capabilities
				Decisions based on my experience is more important than using SCAs (2)				
Behavioural Regulation	3	31	3	I do (11)/ don't (1) have strategies to monitor changes in patients’ health status	21 (68)	5 (16)	5 (16)	Assessing readiness for change; Intentional planning behaviour
				It would help if SCAs were: more available and understandable (1), specifically designed for chiropractic (1), summarized in one that is adopted and widespread (1), computerized records to ease tracking (1), and clinicians use tools to monitor pain and disability (1)				
				I have (10)/ don't have (4) a clear plan under what circumstances I will use SCA in my practice				
Skills	4	53	4	I have (8)/ haven’t (5) been trained to use SCA	34 (64)	6 (11)	13 (25)	Clinical training; Clinician-Patient and clinician –clinician communication skill
				I feel that I have the necessary skills to use SCA (12)				
				Skills required to treat patients with high risk of disability are: ability to screen, good communication, psychosocial training, teamwork, and strong training (12), not sure (1), no course required (1)				
				Communication skills are extremely important for the management of LBP patients using SCA (14)				
Intention	1	16	2	I will (9)/ won't (4) manage all of the next 10 patients using SCA	12 (75)	4 (25)	0	Majority will manage patients using SCAs
				I would manage only who needs SCA (3)				
Goals	1	13	1	The goal of managing NSLBP patients with SCA is not incompatible with achieving another objective. (13)	13 (100)	0	0	Compatibility with achieving other objectives
Memory, Attention & Decision	2	24	3	Deciding if a patient should be managed using SCA is easy (11)	21 (87.5)	3 (12.5)	0	Ease of decision
				The rule of thumb I use is: the clinical presentation of the patient (7), guidelines (2), simplicity (1)				
				I do not use a rule of thumb (3)				
Reinforcement	1	13	1	I would manage NSLBP most of the time using the SCA because rewards are greater and patients are satisfied. (13)	13 (100)	0	0	Better outcomes reinforce the use of SCAs
Environmental Context and Resources	3	46	5	Barriers to using SCA include lack of time and training; seeing fewer patients; and cost. (13)	23 (50)	21 (46)	2 (4)	Environmental resources
				Facilitators to using SCA include having: certified colleague in the team and simplicity (3)				
				No barriers to using SCA. (2)				
				Onsite rehabilitation may be required (6)/ not required (8)				
				There are (11)/ no (3) resources available that help me manage patients using the SCA				
Social Influences	4	51	4	I would (8)/ would not (5) consider consulting more experienced practitioners	32 (63)	17 (33)	2 (4)	Influence of colleagues and researchers ; psychological cases influence decision
				The views of other researchers influence (10)/ don't influence (2)/ may or may not influence (1) my decision to manage patients using SCAs.				
				Having an acute patient in apparent distress would (6)/ wouldn't (5)/ not sure if would (1) influence my decision to manage such patients using the SCA.				
				Having a chronic patient with important psychological overlay would (4)/ wouldn't (9) influence my decision to manage with SCA.				
Optimism	1	12	1	I am generally optimistic regarding the added value of using SCA in my daily practice. (12)	12 (100)	0	0	Positive attitude
Social Professional identity	2	26	2	I consider (12)/ don't consider (1) using SCA to be part of my work as a chiropractor.	25 (96)	1 (4)	0	Professional role; Professional agreement
				I think it is appropriate that my role should include managing patients with non-specific LBP using the SCA. (13)				

^a^Statements perceived to increase use of stratified care approaches (facilitators). ^b^ Statements perceived to reduce use of stratified care approaches (barriers). ^c^ Statements perceived to neither increase/decrease the use of stratified care approaches. *SCA* stratified care approach, *LBP* low back pain

## Domain-specific themes

In this section, we present the specific beliefs and corresponding themes underpinning two key theoretical domains shared by both physiotherapists and chiropractors (*Environmental Context and Resources* and *Social Influences*), followed by the key domains for each discipline.

### Shared domains by physiotherapists and chiropractors

#### Environmental context and resources

##### Physiotherapists

Fifty-two statements concerned *Environmental Context and Resources* domain. Several barriers mentioned regarding the use of stratified care approaches, including: lack of time, cost, other colleagues, lack of expertise, patient preference, and language and unmotivated patients. In contrast, six statements mentioned the facilitators to using stratified care approaches: fewer sessions required, having a private room, autonomy, teamwork and support from management. Nearly all (13/14) physiotherapists indicated that onsite rehabilitation equipment is not required. Moreover, all physiotherapists mentioned that there are resources available that help manage non-specific LBP patients using stratified care approaches.

##### Chiropractors

Forty-six statements were related to the *Environmental Context and Resources* domain*.* Thirteen chiropractors mentioned the barriers to using stratified care approaches were: a lack of time; seeing fewer patients, and cost. Two chiropractors reported that no barriers existed. Three chiropractors mentioned facilitators to using stratified care approaches including having a certified colleague in the team and the simplicity of the stratified care approaches. More than half of the chiropractors indicated that there is no onsite rehabilitation equipment (low tech or high tech) or access to specialized care required to implement stratified care approaches in their practice. While six participants indicated that onsite rehabilitation equipment (physical space, and physical model for demonstration) and having access to specialized care may be required to implement stratified care approaches.

On the other hand, 11 chiropractors mentioned that there are resources available to help manage non-specific LBP patients using the stratified care approach, such as: online websites, educational pamphlets, YouTube videos, MacKenzie book, and training manual of the CORE back tool. However, three chiropractors noted the unavailability of such resources in their clinics.

#### Social influences

##### Physiotherapists

Fifty-eight statements concerned *Social Influences* domain. Nine physiotherapists said they would consult more experienced practitioners if they needed help, while four physiotherapists would not. For example, some physiotherapists said they often referred difficult cases to, and sought advice from, colleagues. The views of other colleagues influenced (*n* = 9) or did not influence (*n* = 7) physiotherapists’ decision to manage patients using stratified care approach. However, the views of researchers influenced their decision to manage patients using care stratified care approach. Thirteen physiotherapists indicated that having an acute LBP patient in apparent distress would not influence their decisions to manage non-specific LBP patients using the stratified care approach. The majority of physiotherapists mentioned that having chronic patients with important psychological overlay would not influence their decision to manage them using a stratified care approach.

##### Chiropractors

Fifty-one statements were about *Social Influences* domain. Eight chiropractors considered consulting other staff but not chiropractic colleagues, while five other chiropractors did not. The majority (10/13) of chiropractors mentioned that the views of researchers influenced their decision to manage patients using stratified care approach, two chiropractors said it did not, and one other said he was unsure. About half of the participants (6/13) said that having an acute LBP patient in apparent distress would influence their decisions to manage non-specific LBP patients using the stratified care approach, five chiropractors said this would not influence their decision, and one was unsure. Nine chiropractors said having a patient with chronic LBP with important psychological overlay would not influence their decision to manage them using stratified care approaches while four considered that it would influence their decision.

#### Key domains only for physiotherapists

##### Goals

Sixteen statements related to the *Goals* domain, including 12 expressing that the goal of managing non-specific LBP patients with stratified care approaches may be incompatible with achieving another objective. In contrast, four statements suggested this was not incompatible.

#### Key domains only for chiropractors

##### Knowledge

All respondents expressed awareness of existing stratified care approaches. Chiropractors reported their understanding of the use of stratified care approaches as follows: the majority said it serves to classify patients into groups to provide effective treatment for each group. One respondent noted that stratified care approaches can streamline different professionals’ work, while another mentioned that there are several types of stratified care approaches. One interviewee considered himself to have limited understanding of how to use stratified care approaches. Most chiropractors (11/13) agreed with the recommended use of stratified care approaches for LBP patients. However, two respondents did not necessarily agree with the recommended use of stratified care approaches. Seven respondents mentioned that they know how to use stratified care approaches to target the management of non-specific LBP patients. Four respondents said although they know how to use stratified care approaches, they don’t routinely use them in their daily practice. Two chiropractors said they did not know how to use stratified care approaches to target the management of non-specific LBP patients.

##### Belief about consequences

Fifty-eight statements related to the *Belief about consequences* domain. Thirty-nine statements highlighted the likely benefits of using stratified care approaches including managing patients and providing evidence-based practice (EBP). Another 12 statements indicated that chiropractors using stratified care approaches expected to see changes in outcomes such as: less pain, better function, faster recovery, higher satisfaction, and less medication. On the contrary, seven statements highlighted the likely disadvantages of using stratified care approaches such as more focus on yellow flags, require memorizing and time consuming.

##### Belief about capabilities

Seventeen statements were related to the *Beliefs about capabilities* domain, including 11 statements expressing the confidence of chiropractors in assessing non-specific LBP patients using stratified care approaches and matching the targeted treatments. However, four statements expressed lacking confidence to use stratified care approach. Two chiropractors considered making decisions based on their experience to be more important than relying on stratified care approaches.

##### Behavioural regulation

A total of 31 statements were coded to the *Behavioural regulation* domain. Chiropractors acknowledged the following strategies to be important for improving patient outcomes: regularly monitoring the patients’ health status and having a clear plan for the circumstances in which they will use stratified care approaches in their practice. However, some (4/13) chiropractors did not have a clear plan under what circumstances they would use stratified care approaches in their practice.

##### Intention

A total of 16 statements were relevant to the *Intention* domain. Nine chiropractors had the intention to manage non-specific LBP patients using stratified care approaches, and four did not. Three other statements considered managing patients with stratified care approaches only for those who needed it.

## Discussion

The perceived benefits of using stratified care approaches to manage non-specific LBP for both the chiropractic and physiotherapy disciplines included empowering patients to self-manage, addressing psychosocial issues, better health outcomes, a more accurate prognosis, implementing EBP and a more uniform practice between clinicians. The lesser reluctance of physiotherapists to routinely use stratified care approaches in clinical practice may be attributed to formal stratified care approaches of undergraduate or postgraduate training compared to chiropractors. Factors perceived as strongly influencing the use of stratified care approaches clustered around three theoretical domains (*Environmental Context and Resources; Social Influences;* and *Goals*) for physiotherapists and seven key domains (*Environmental Context and Resources; Beliefs about Capabilities; Social Influences; Intentions; Knowledge; Behavioural Regulation;* and *Beliefs about Consequences*) for chiropractors. Interestingly, the two domains shared by both disciplines (*Environmental Context and Resources* and *Social Influences*) were frequently reported in a review of exploratory studies underpinned by the TDF among different health disciplines across a range of behaviours [53]. For instance, barriers observed in our study such as lack of time, cost, and other colleagues in the same practice who are unfamiliar with stratified care approaches (*Environmental Context and Resources*) were also concerns raised by UK physiotherapists [54]. Our findings are also consistent with other studies showing that across different professions, clinicians are generally more receptive to evidence communicated by their peers (*Social Influences*) than research evidence and clinical practice guidelines [13, 15, 39, 55–73]. Nonetheless, about one third of our participants from both disciplines indicated that they would not consider consulting more experienced peers, and most chiropractors indicated that the views of researchers would likely influence their decision to manage patients using stratified care approaches. Interestingly, the decision of physiotherapists to manage acute/chronic LBP patients in apparent distress using the stratified care approaches appeared to be easier (less influenced) than for chiropractors, possibly because physiotherapists receive more undergraduate training in using psychological interventions [55].

For the majority of participating physiotherapists, the goal of managing non-specific LBP patients with stratified care approaches (Goals domain) may be incompatible with dealing with others’ goals such as: the clinic owner wishing keep the patients for longer time, patients’ beliefs or attitudes (resistance to using stratified care approaches and fear of movement) and clinical presentation (pain level and psychological overlay). This may be explained by the fact that physiotherapists tend to work in group practices and need to adhere to specific rules set by the clinic owner [74]. In contrast, half of chiropractors in Canada work as solo practitioners [75, 76], possibly explaining why none of the interviewed chiropractors mentioned that incompatibility of stratified care approaches was an issue.

On the other hand, five additional theoretical domains (*Beliefs about Capabilities; Intentions; Knowledge; Behavioural Regulation;* and *Beliefs about Consequences*) were deemed important for chiropractors. While two-thirds of chiropractors felt confident in assessing non-specific LBP patients using stratified care approaches and matching the targeted treatments (*Beliefs about Capabilities*) others considered that their experience was more important in identifying the most appropriate treatment than using stratified care approaches. This is in line with previous findings where a small percentage of chiropractors admitted they preferred relying on their personal experience to guide treatment for neck pain [52].

Chiropractors expressed high *Intentions* of managing non-specific LBP patients using stratified care approaches. However, about one third of chiropractors said they would not use stratified care approaches to manage non-specific LBP patients, with some concerns raised regarding certain stratified care approaches such as STarT Back Tool [32] because chiropractors were not involved in the original research, unlike the physiotherapy profession [77]. This is in line with a systematic review of the determinants of professionals’ intentions and behaviours which concluded that professional role and identity is an essential determinant of intention [78]. Another review showed that there are few studies regarding the use of stratified care approaches like Start Back Tool in chiropractic patients [79].

Chiropractors expressed their awareness and understanding of stratified care approaches in terms of *Knowledge*. However, about half of the chiropractors either did not use stratified care approaches or did not know how to apply them in the management of non-specific LBP patients. This might explain why one-third of chiropractors were not confident in using stratified care approaches and considered their experience to be more important than using stratified care approaches. Previous work showed that chiropractors usually appreciated researchers’ efforts but felt it was not applicable to their daily clinical practice [80–82].

About two-thirds of chiropractors mentioned that they have a clear plan under what circumstances they will use stratified care approaches in their practice (*Behavioural Regulation* domain). Statements relating to *Beliefs about Consequences* shed some light on some of the perceived disadvantages of using stratified care approaches in chiropractic practice, including: stratified care approaches mistakenly identify patients and lead to overtreatment, limited patient assessment, are too simplistic, do not cover lifestyle factors, are more focused on yellow flags (i.e., risk of delayed recovery), are not widely used, and do not speed up recovery.

### Strengths and limitations

To our knowledge, this is the first study to compare physiotherapists’ and chiropractors’ beliefs and attitudes toward using stratified care approaches for managing non-specific LBP. Our findings provide valuable insight and form the basis for proceeding with designing and evaluating tailored implementation strategies to increase the use of stratified care approach. One of the strengths of this study was the rigor of the coding process that was undertaken for developing themes. Nonetheless, our study has several limitations. The sample size was small but appropriate for a qualitative study underpinned by the TDF [43]. Further, the proportion of female chiropractors was smaller than the national average [83]. Similar themes started to recur by the third interview, and no new themes emerged after the 11th interview for chiropractors and the 12th interview for physiotherapists. Thus, it is unlikely that recruiting more participants would have changed the overall balance between different factors likely to increase or decrease our targeted behaviour. On the other hand, self-reported confidence in using stratified care approaches for managing non-specific LBP was quite high among our interviewees, possibly reflecting volunteer bias. Other important barriers would likely have been identified if patients with non-specific LBP had been included in the study. Patients’ beliefs, preferences, and expectations may largely impact professionals’ practices and can be a barrier to the appropriate use of stratified care approach [84]. Therefore, it is important for future research to evaluate patients’ attitudes, beliefs, and experiences on the use of stratified care approaches.

## Conclusion

The findings of this study indicate that both physiotherapists and chiropractors were aware of the existence of different stratified care approaches. Different barriers and facilitators were identified for each discipline. These determinants reflect the nature and type of practices, as well the prior training. Nonetheless, participants from both disciplines generally agreed about the benefits of stratified care approaches for managing non-specific LBP. These included empowering patients to self-manage, implementing EBP, considering psychosocial risk factors, a more uniform practice between clinicians, and better health outcomes. Determinants identified may inform the design of a theory-based KT intervention to increase the use of selected stratified care approaches to manage non-specific LBP patients.

## Supplementary information

**Additional file 1.** Semi-Structured Interview Guide for Clinician (chiropractors and physical therapists) Interviews.

**Additional file 2.** Findings of relevant domains together with illustrative quotes for physiotherapists.

**Additional file 3.** Findings of relevant domains together with illustrative quotes for chiropractors.

## Data Availability

The datasets generated during and/or analysed during the current study are available from the corresponding author on reasonable request.

## Abbreviations

EBP: Evidence-based practice
Non-specific LBP: Non-Specific Low Back Pain
TDF: Theoretical Domains Framework

## References

[1] Vos T, Flaxman AD, Naghavi M, Lozano R, Michaud C, Ezzati M (2012). Years lived with disability (YLDs) for 1160 sequelae of 289 diseases and injuries 1990-2010: a systematic analysis for the global burden of disease study 2010. Lancet.

[2] Dagenais S, Caro J, Haldeman S (2008). A systematic review of low back pain cost of illness studies in the United States and internationally. Spine J.

[3] Andersson GB (1999). Epidemiological features of chronic low-back pain. Lancet.

[4] Frank AO, De Souza LH (2001). Conservative management of low back pain. Int J Clin Pract.

[5] Scheermesser M, Bachmann S, Schamann A, Oesch P, Kool J (2012). A qualitative study on the role of cultural background in patients' perspectives on rehabilitation. BMC Musculoskelet Disord.

[6] Horng YS, Hwang YH, Wu HC, Liang HW, Mhe YJ, Twu FC (2005). Predicting health-related quality of life in patients with low back pain. Spine (Phila Pa 1976).

[7] Di Iorio A, Abate M, Guralnik JM, Bandinelli S, Cecchi F, Cherubini A (2007). From chronic low back pain to disability, a multifactorial mediated pathway: the InCHIANTI study. Spine (Phila Pa 1976).

[8] Widanarko B, Legg S, Stevenson M, Devereux J, Jones G (2013). Prevalence of low back symptoms and its consequences in relation to occupational group. Am J Ind Med.

[9] GBD 2016 Disease and Injury Incidence and Prevalence Collaborators. Global, regional, and national incidence, prevalence, and years lived with disability for 328 diseases and injuries for 195 countries, 1990–2016: a systematic analysis for the Global Burden of Disease Study 2016. Lancet. 2017;390(10100):1211–59. https://www.ncbi.nlm.nih.gov/pubmed/28919117. 10.1016/S0140-6736(17)32154-2 PMC5605509 28919117

[10] Freburger JK, Holmes GM, Agans RP, Jackman AM, Darter JD, Wallace AS (2009). The rising prevalence of chronic low back pain. Arch Intern Med.

[11] Katz JN (2006). Lumbar disc disorders and low-back pain: socioeconomic factors and consequences. J Bone Joint Surg Am.

[12] Church J, Schneider M, Shipka P, Triska O, Smith D, LS L (2002). Review of current knowledge on the effectiveness and cost effectiveness of treatments for low back conditions.

[13] Manek NJ, MacGregor AJ (2005). Epidemiology of back disorders: prevalence, risk factors, and prognosis. Curr Opin Rheumatol.

[14] van Tulder M, Koes B, Bombardier C (2002). Low back pain. Best Pract Res Clin Rheumatol.

[15] Kent P, Keating J (2004). Do primary-care clinicians think that nonspecific low back pain is one condition?. Spine (Phila Pa 1976).

[16] Rinkus KM, Knaub MA (2008). Clinical and diagnostic evaluation of low Back Pain. Semin Spine Surg.

[17] Foster NE (2011). Barriers and progress in the treatment of low back pain. BMC Med.

[18] Foster NE, Hill JC, Hay EM (2011). Subgrouping patients with low back pain in primary care: are we getting any better at it?. Man Ther.

[19] Billis EV, McCarthy CJ, Oldham JA (2007). Subclassification of low back pain: a cross-country comparison. Eur Spine J.

[20] Riddle DL (1998). Classification and low back pain: a review of the literature and critical analysis of selected systems. Phys Ther.

[21] Fairbank J, Gwilym SE, France JC, Daffner SD, Dettori J, Hermsmeyer J (2011). The role of classification of chronic low back pain. Spine (Phila Pa 1976).

[22] Fourney DR, Dettori JR, Hall H, Hartl R, McGirt MJ, Daubs MD (2011). A systematic review of clinical pathways for lower back pain and introduction of the Saskatchewan spine pathway. Spine (Phila Pa 1976).

[23] Karayannis NV, Jull GA, Hodges PW (2012). Physiotherapy movement based classification approaches to low back pain: comparison of subgroups through review and developer/expert survey. BMC Musculoskelet Disord.

[24] Delitto A, George SZ, Van Dillen LR, Whitman JM, Sowa G, Shekelle P (2012). Low back pain. Clinical practice guidelines linked to the international classification of functioning, disability, and health from the Orthopaedic section of the American Physical Therapy Association. J Orthop Sports Phys Ther..

[25] McKenzie R. The lumbar spine: mechanical diagnosis and therapy. Waikanae, New Zealand: Spinal Publication; 1981.

[26] Savigny P, Watson P, Underwood M (2009). Early management of persistent non-specific low back pain: summary of NICE guidance. BMJ.

[27] Chou R, Deyo R, Friedly J, Skelly A, Hashimoto R, Weimer M, et al. Noninvasive treatments for low Back Pain. Comparative effectiveness review no. 169. (prepared by the Pacific northwest evidence-based practice center under contract no. 290–2012-00014-I.) AHRQ publication no. 16-EHC004-EF. Rockville: Agency for Healthcare Research and Quality; 2016. www.effectivehealthcare.ahrq.gov/reports/final.cfm. Accessed 23 Nov 2018.

[28] Ferguson FC, Brownlee M, Webster V (2008). A Delphi study investigating consensus among expert physiotherapists in relation to the management of low back pain. Musculoskelet Care.

[29] Mikhail C, Korner-Bitensky N, Rossignol M, Dumas JP (2005). Physical therapists' use of interventions with high evidence of effectiveness in the management of a hypothetical typical patient with acute low back pain. Phys Ther.

[30] Pincus T, Vogel S, Breen A, Foster N, Underwood M (2006). Persistent back pain--why do physical therapy clinicians continue treatment? A mixed methods study of chiropractors, osteopaths and physiotherapists. Eur J Pain.

[31] Hill JC, Whitehurst DG, Lewis M, Bryan S, Dunn KM, Foster NE (2011). Comparison of stratified primary care management for low Back pain with current best practice (STarT Back): a randomised controlled trial. Lancet.

[32] Hill JC, Dunn KM, Lewis M, Mullis R, Main CJ, Foster NE (2008). A primary care back pain screening tool: identifying patient subgroups for initial treatment. Arthritis Rheum.

[33] Lam OT, Strenger DM, Chan-Fee M, Pham PT, Preuss RA, Robbins SM (2018). Effectiveness of the McKenzie method of mechanical diagnosis and therapy for treating low Back Pain: literature review with meta-analysis. J Orthop Sports Phys Ther.

[34] Cane J, O'Connor D, Michie S (2012). Validation of the theoretical domains framework for use in behaviour change and implementation research. Implement Sci.

[35] Michie S, Johnston M, Abraham C, Lawton R, Parker D, Walker A (2005). Making psychological theory useful for implementing evidence based practice: a consensus approach. Qual Saf Health Care.

[36] Dyson J, Rebecca L, Cath J, Francine C (2011). Does the use of a theoretical approach tell us more about hand hygiene behaviour? The barriers and levers to hand hygiene. J Infect Prev.

[37] Michie S, Pilling S, Garety P, Whitty P, Eccles MP, Johnston M (2007). Difficulties implementing a mental health guideline: an exploratory investigation using psychological theory. Implement Sci.

[38] McKenzie JE, French SD, O'Connor DA, Grimshaw JM, Mortimer D, Michie S (2008). IMPLEmenting a clinical practice guideline for acute low back pain evidence-based manageMENT in general practice (IMPLEMENT): cluster randomised controlled trial study protocol. Implement Sci.

[39] McKenzie JE, O'Connor DA, Page MJ, Mortimer DS, French SD, Walker BF (2010). Improving the care for people with acute low-back pain by allied health professionals (the ALIGN trial): A cluster randomised trial protocol. Implement Sci.

[40] Bussieres AE, Patey AM, Francis JJ, Sales AE, Grimshaw JM, Brouwers M (2012). Identifying factors likely to influence compliance with diagnostic imaging guideline recommendations for spine disorders among chiropractors in North America: a focus group study using the theoretical domains framework. Implement Sci.

[41] French SD, Green SE, O'Connor DA, McKenzie JE, Francis JJ, Michie S (2012). Developing theory-informed behaviour change interventions to implement evidence into practice: a systematic approach using the Theoretical Domains Framework. Implement Sci.

[42] Amemori M, Korhonen T, Kinnunen T, Michie S, Murtomaa H (2011). Enhancing implementation of tobacco use prevention and cessation counselling guideline among dental providers: a cluster randomised controlled trial. Implement Sci.

[43] Francis JJ, Johnston M, Robertson C, Glidewell L, Entwistle V, Eccles MP (2010). What is an adequate sample size? Operationalising data saturation for theory-based interview studies. Psychol Health.

[44] Atkins L, Francis J, Islam R, O'Connor D, Patey A, Ivers N (2017). A guide to using the theoretical domains framework of behaviour change to investigate implementation problems. Implement Sci.

[45] Espeland A, Baerheim A (2003). Factors affecting general practitioners' decisions about plain radiography for back pain: implications for classification of guideline barriers--a qualitative study. BMC Health Serv Res.

[46] Bishop A, Thomas E, Foster NE (2007). Health care practitioners' attitudes and beliefs about low back pain: a systematic search and critical review of available measurement tools. Pain.

[47] Pincus T, Foster NE, Vogel S, Santos R, Breen A, Underwood M (2007). Attitudes to back pain amongst musculoskeletal practitioners: a comparison of professional groups and practice settings using the ABS-mp. Man Ther.

[48] Biggs L, Mierau D, Hay D (2002). Measuring philosophy: a philosophy index. J Can Chiropr Association.

[49] Francis JJ, Stockton C, Eccles MP, Johnston M, Cuthbertson BH, Grimshaw JM (2009). Evidence-based selection of theories for designing behaviour change interventions: using methods based on theoretical construct domains to understand clinicians' blood transfusion behaviour. Br J Health Psychol.

[50] Francis JJ, Tinmouth A, Stanworth SJ, Grimshaw JM, Johnston M, Hyde C (2009). Using theories of behaviour to understand transfusion prescribing in three clinical contexts in two countries: development work for an implementation trial. Implement Sci.

[51] Hsieh HF, Shannon SE (2005). Three approaches to qualitative content analysis. Qual Health Res.

[52] Bussieres AE, Al Zoubi F, Quon JA, Ahmed S, Thomas A, Stuber K (2015). Fast tracking the design of theory-based KT interventions through a consensus process. Implement Sci.

[53] Mosavianpour M, Sarmast HH, Kissoon N, Collet JP (2016). Theoretical domains framework to assess barriers to change for planning health care quality interventions: a systematic literature review. J Multidiscip Healthc.

[54] Sheeran L, Coales P, Sparkes V (2015). Clinical challenges of classification based targeted therapies for non-specific low back pain: what do physiotherapy practitioners and managers think?. Man Ther.

[55] Alexanders J, Anderson A, Henderson S (2015). Musculoskeletal physiotherapists' use of psychological interventions: a systematic review of therapists' perceptions and practice. Physiother.

[56] Borrell C, Muntaner C, Benach J, Artazcoz L (2004). Social class and self-reported health status among men and women: what is the role of work organisation, household material standards and household labour?. Soc Sci Med.

[57] Bot SD, Terwee CB, van der Windt DA, Bouter LM, Dekker J, de Vet HC (2004). Clinimetric evaluation of shoulder disability questionnaires: a systematic review of the literature. Ann Rheum Dis.

[58] Briggs MS, Givens DL, Schmitt LC, Taylor CA (2013). Relations of C-reactive protein and obesity to the prevalence and the odds of reporting low back pain. Arch Phys Med Rehabil.

[59] Burton AK, Balague F, Cardon G, Eriksen HR, Henrotin Y, Lahad A (2006). Chapter 2. European guidelines for prevention in low back pain : November 2004. Eur Spine J.

[60] de Boer AG, van Lanschot JJ, Stalmeier PF, van Sandick JW, Hulscher JB, de Haes JC (2004). Is a single-item visual analogue scale as valid, reliable and responsive as multi-item scales in measuring quality of life?. Qual Life Res.

[61] Elliott TE, Renier CM, Palcher JA (2003). Chronic pain, depression, and quality of life: correlations and predictive value of the SF-36. Pain Med.

[62] Lame IE, Peters ML, Vlaeyen JW, Kleef M, Patijn J (2005). Quality of life in chronic pain is more associated with beliefs about pain, than with pain intensity. Eur J Pain.

[63] Luo X, Pietrobon R, Sun SX, Liu GG, Hey L (2004). Estimates and patterns of direct health care expenditures among individuals with back pain in the United States. Spine (Phila Pa 1976).

[64] Pain K, Magill-Evans J, Darrah J, Hagler P, Warren S (2004). Effects of profession and facility type on research utilization by rehabilitation professionals. J Allied Health.

[65] Rapoport J, Jacobs P, Bell NR, Klarenbach S (2004). Refining the measurement of the economic burden of chronic diseases in Canada. Chronic Dis Can.

[66] Rudwaleit M, van der Heijde D, Khan MA, Braun J, Sieper J (2004). How to diagnose axial spondyloarthritis early. Ann Rheum Dis.

[67] Steenstra IA, Verbeek JH, Heymans MW, Bongers PM (2005). Prognostic factors for duration of sick leave in patients sick listed with acute low back pain: a systematic review of the literature. Occup Environ Med.

[68] Woolf AD, Pfleger B (2003). Burden of major musculoskeletal conditions. Bull World Health Organ.

[69] Gerrish K, Clayton J (2004). Promoting evidence-based practice: an organizational approach. J Nurs Manag.

[70] Hundley V, Milne J, Leighton-Beck L, Graham W, Fitzmaurice A (2000). Raising research awareness among midwives and nurses: does it work?. J Adv Nurs.

[71] McAlister FA, Graham I, Karr GW, Laupacis A (1999). Evidence-based medicine and the practicing clinician. J Gen Intern Med.

[72] Suter E, Verhoef M, O'Beirne M (2004). Assessment of the information needs and use of information resources on complementary and alternative medicine by Alberta family physicians. Clin Invest Med.

[73] Jette DU, Bacon K, Batty C, Carlson M, Ferland A, Hemingway RD (2003). Evidence-based practice: beliefs, attitudes, knowledge, and behaviors of physical therapists. Phys Ther.

[74] Canadian Institute for Health Information, Physiotherapists in Canada, 2009. Ottawa: CIHI; 2010. https://www.cptbc.org/pdf/CIHIReport.PTinCanada.2009.pdf. Accessed 15 Nov 2018.

[75] Lawrence DJ, Meeker WC (2007). Chiropractic and CAM Utilization: A Descriptive Review. Chiropr Osteopat.

[76] Bureau of Labor Statistics - U.S. Department of Labor. Occupational Outlook Handbook - Chiropractors. 2016–17 Edition. 2016. http://www.bls.gov/ooh/healthcare/chiropractors.htm. Accessed 18 Dec 2018.

[77] Al Zoubi FM, Eilayyan O, Mayo NE, Bussieres AE (2017). Evaluation of cross-cultural adaptation and measurement properties of STarT Back screening tool: a systematic review. J Manip Physiol Ther.

[78] Godin G, Belanger-Gravel A, Eccles M, Grimshaw J (2008). Healthcare professionals' intentions and behaviours: a systematic review of studies based on social cognitive theories. Implement Sci.

[79] Khan Y (2017). The STarT back tool in chiropractic practice: a narrative review. Chiropr Man Therap.

[80] Hall G (2011). Attitudes of chiropractors to evidence-based practice and how this compares to other healthcare professionals: a qualitative study. Clin Chiropr.

[81] Thomas A, Law M (2013). Research utilization and evidence-based practice in occupational therapy: a scoping study. Am J Occup Ther.

[82] Bussieres AE, Al Zoubi F, Stuber K, French SD, Boruff J, Corrigan J (2016). Evidence-based practice, research utilization, and knowledge translation in chiropractic: a scoping review. BMC Complement Altern Med.

[83] Canadian Institutes for Health Information (CIHI). Canada’s Health Care Providers: Provincial Profiles, 2008 to 2017 — Data Tables 2019. https://www.cihi.ca/en/canadas-health-care-providers-provincial-profiles-2008-to-2017-data-tables. Accessed 15 Jan 2019.

[84] Abresch RT, Carter GT, Han JJ, McDonald CM (2009). New clinical end points in rehabilitation medicine: tools for measuring quality of life. Am J Hosp Palliat Care.

