# Supplementary material for: Professional barriers and facilitators to using stratified care approaches for managing non-specific low back pain: a qualitative study with Canadian physiotherapists and chiropractors
Source: Chiropr Man Therap. 2019 Dec 13;27:68. doi: 10.1186/s12998-019-0286-3 (PMC6909494; doi:10.1186/s12998-019-0286-3)
Supplement: Supplementary file 1 — Additional file 1. Semi-Structured Interview Guide for Clinician (chiropractors and physical therapists) Interviews. [file 12998_2019_286_MOESM1_ESM.docx]

**Additional file 1: Semi-Structured Interview Guide for Clinician (chiropractors and physical therapists) Interviews**

***‘‘Professional barriers and facilitators to using stratified care approaches for non-specific low back pain’’***

**Interview Guide**

I’d like to begin by asking you to state your code of the participant slowly for the benefit of the person who will be transcribing the interview.

Could you describe *how you manage new patients with low back pain (LBP) in your office? By that I mean your usual clinical routine once the patient is with you in the examining room, such as case history questions, examination procedures, and whether you manage acute or chronic LBP patients using stratified care approaches (SCA) such as Mechanical Diagnosis and Treatment (McKenzie), Treatment-Based Classification (Delitto), Motor control impairments (O’ Sullivan), Movement System Impairment Syndromes (Sahrmann), STarT Back Screening Tool as part of your management strategy.*

*(Prompts: What do you feel you do well? What do you feel you could do better? Do you use the SCAs in your approach with patients? Why or why not?)*

*Thank you.*

I will start the interview by asking you some demographic information:

**Part A: Socio-Demographic Information**

**1.** Age:

**2.** Sex: ☐ Male ☐ Female

**3.** What is the highest level of education that you have completed?

☐ Diploma Program ☐ Undergraduate Degree ☐ Master Degree

☐ PhD Degree

☐ Other (please specify): _____________________

**4.** How many years of clinical experience you have? __________________

**5.** What is your current employment status?

☐ Full-time employed (more than 16 hours in patient- contact hours)

☐ Part-time employed (less than 16 hours in patient- contact hours)

☐ Not employed

**6.** Practice type /workplace?

☐ Solo practice

☐ Group practice

☐ Other

**7.** Clinical setting:

☐ Private

☐ Multidisciplinary health care center

☐ Rehabilitation center

☐ Hospitals

**8.** Socioeconomic status (SES) of your patients (on average):

☐ Mostly high SES

☐ Middle SES

☐ Mostly low SES

**9.** Where do you practice?

☐ Urban area

☐ Suburban area

☐ Rural area

**10.** On average, how many patients (new and regular visit) per week do you see **in total**?

**11.** On average, how many **patients** (new and regular visit) with **low back pain** do you see per week? __________________________________

**12.** On average, how many **NEW cases** of low back pain, do you see per week? __________________________________

Now for the rest of the interview, I have more specific questions. Some may seem repetitive, but please bear with me. We would like to understand how clinicians make clinical decisions about managing patients with low back pain. I may also seek clarification during the interview via probing questions such as: *‘What do you mean’; ‘Would you explain this’; ‘What were you thinking at the time’; ’Walk me through your experience’, ‘What skills do you need?’; ‘How and why do you use it?’* I would like to encourage you to say as much as you like, be as detailed and descriptive as you can.

***The key recommendations from the Low Back Pain Guidelines and recent literature are:***

- Current evidence suggests that the use of stratified care approaches (SCA) can improve patient outcomes in the primary care setting.
- A recent clinical practice guideline [[17](#_ENREF_17)] suggested using *SCA* for non-specific LBP.
- In addition, some studies have shown that the use of the *SCA* in clinical practice appears to be associated with lower costs and higher patient satisfaction.

**For the purpose of this interview:**

- **Stratified care approaches are designed to assist clinicians with matching specific treatments strategy to the patient’s clinical presentation. These approaches are sometimes referred to as classification systems.**
- **‘Non-specific low back pain’ refers to patients with uncomplicated LBP that varies with time and activity with no neurologic deficits, fractures or indicators of potentially serious pathologies (i.e. red flags).**

**If you are ready, I will now ask you specific questions on this topic.**

**Knowledge**

1. Are you aware of any existing stratified care approaches (SCA) to help manage low back pain patients?
2. Please tell me about your understanding about the use of *SCA* to manage non-specific LBP? *(Prompt: Did reading the proposed guideline and scientific papers improve your understanding?)*
3. Do you agree with the recommended use of *SCA* for managing LBP patients [[17-20](#_ENREF_17)] ? *(Prompt: What alternative, if any, would help you conform with recommended care?)*
4. Do you know how to use *SCA to target the management of non-specific LBP patients*? *(Prompt: Could you explain it to me?)*

*(Prompts: How do you use it? Why do you use it? What do you think of it)?*

*(Prompt: What alternative, if any, would help you conform with recommended care?)*

**Beliefs about consequences (Anticipated outcome/attitude)**

1. What are the possible benefits of managing a patient using SCA and its targeted treatment approaches for non-specific LBP?

*(Prompt: Better patient health outcomes? Shorter recovery times?)*

1. What are the potential disadvantages of managing patients with non-specific LBP without using the *SCAs*?

*(Prompts: 1) Longer recovery time 2) Patient preference and satisfaction)*

1. What outcome do you expect if you manage a patient with non-specific LBP using *SCA* and its targeted treatment approaches?

**Beliefs about capabilities (self-efficacy)**

1. How confident are you in assessing non-specific LBP patients using *SCA* and determining the targeted treatment/s?

*(Prompt: How easy or difficult is this)?*

**Behavioural regulation**

1. Do you monitor changes in non-specific LBP patients’ health status after using *SCA*? How?

*(Prompts: After treating non-specific LBP patients, what is the usual outcome (do patients improve/deteriorate?)*

1. What could help you manage non-specific LBP patients using the *SCA*?
2. Do you have a clear plan under what circumstances you will use *SCA* in your practice? What if the patients are not motivated? Do you think having one would help?

**Skills**

1. Have you been trained in using any of the *SCA* approaches?
2. Do you feel that you have the necessary skills to use *SCA*? If not, what new skills would you require to be able to manage non-specific LBP patients using the *SCA?*

*(Prompts: Additional training, counselling skills /communication techniques, continuing education, educational material, online information.)*

1. What skills are required to manage patients at high risk of disability *using SCA*?

*(Prompt: Do they have the skills to manage the functional issues of these patients?)*

1. What do you think about the importance of communication skills for the management of LBP patients using the *SCA*?

*(Prompt: Why?)*

**Intention**

1. Of the next 10 non-specific LBP patients, how many of them will you manage using an SCA approach

**Goals**

1. Would the goal of managing non-specific LBP patient with *SCA* be incompatible with achieving some other objective? (i.e., anything else that you want to do or achieve that might interfere with this).

*(Prompt: Slower recovery time or discharge)*

**Memory, Attention and Decision Processes**

1. How easy or difficult is it to decide if a patient should be managed using *SCA* or not?
2. What rules of thumb, if any, do you use to reach a decision about the best *SCA* that should be used?

*(Prompts: Decision rules, guidelines…)?*

**Reinforcement**

1. On a scale where one means ‘none of the time’ and five means ‘most of the time’’, to what extent would you manage non-specific LBP patients using the *SCA,* if the rewards (e.g., better patient satisfaction, less patient discomfort, etc.) were greater than when not using a SCA? Can you explain why you gave this score?

*(Prompts: 1) Longer recovery time 2) Patient preference and satisfaction)*

**Environmental context and resources (environmental constraints)**

1. Are there any factors in your practice likely to either help or prevent you from using the *SCA* for patients with non-specific LBP?

*(Prompts: Running late, having a staff shortage on any particular day, the management or staff of the organization/practice, not having the expertise to provide the appropriate care to the high risk patients)*

1. Do you think having or not having onsite rehabilitation equipment (low tech or high tech) or access to specialized care may influence your decision to manage non-specific LBP patients using *SCA*?
2. Are there any resources available that you could use to help you manage patients with non-specific LBP patients using the *SCA*?

*(Prompt: Information pamphlets, posters, or online tools to inform patients about the benefits of matching specific intervention based on the interpreted symptoms)?*

**Social influences (Norms)**

1. Are there instances where you may consider consulting other people (such as colleagues or staff) for their opinion regarding the need to use *SCA*? Why?

*(Prompts: Peers, managers, other professional groups, patients).*

1. How do the views of other colleagues or researchers influence your decision to manage patients using *SCA*?

**Emotion (Stress/concerns)**

1. Is seeing acute LBP patients in apparent distress likely to influence your decision to manage such patients using the *SCA*?

*(Prompts: Why or why not?)*

1. Does seeing a patient with chronic LBP, particularly if there is important psychological overlay such as depression or anxiety, likely to influence your decision to manage such patients using *SCA*?

*(Prompts: Why or why not?)*

**Optimism**

1. Are you generally optimistic regarding the added value of using SCA, in your daily practice?

**Social/professional role and identity (self-standards)**

1. Do you consider using *SCA* is part of your work as a clinician (PT/ DC)? *(Prompts: Why or why not?)*
2. Do you think it is appropriate that your role should include managing patients with non-specific LBP using the *SCA*?

*(Prompts: Why or why not?)*

Those are all of the questions I have for you today. Has anything else occurred to you about this topic that we haven’t asked about? Are there any other thoughts you wish to convey on this topic?
