# Supplementary material for: Professional barriers and facilitators to using stratified care approaches for managing non-specific low back pain: a qualitative study with Canadian physiotherapists and chiropractors
Source: Chiropr Man Therap. 2019 Dec 13;27:68. doi: 10.1186/s12998-019-0286-3 (PMC6909494; doi:10.1186/s12998-019-0286-3)
Supplement: Supplementary file 2 — Additional file 2. Findings of relevant domains together with illustrative quotes for physiotherapists. [file 12998_2019_286_MOESM2_ESM.docx]

**Additional file 2: Findings of relevant domains together with illustrative quotes for physiotherapists**

| **TDF Domains** | **Specific beliefs** | **Selected statements** |
| --- | --- | --- |
| *Knowledge* | I am aware of existing SCA. | *"I’m aware of Mackenzie, of Treatment Based, I’m aware of Peter O’Sullivan, Shirley Sahrmann, there are several of them that I’m fairly familiar with". (ON2)* |
|  | My understanding about the use of SCA is to classify patients into groups to provide effective treatment for each group. | *"If you can classify a patient in the correct classification then you will have an appropriate diagnosis and therefore your treatment plan can follow suit and you will get positive results because you have assessed and classified them properly". (AB4)* |
|  | I agree with the recommended use of SCA for LBP patients. | *"Yes I do agree with it". (ON4)* |
|  | I know how to use SCA to target the management of non-specific LBP patients. | *"I think I do! I had to go through the certification process so they test you on that". (ON3)* |
| *Belief about consequences* | I believe the benefits of using SCA include empowering patients to self-manage, more accurate assessment, better matching of treatment, minimizing visits and costs, increasing self-efficacy, less passive treatment. | *"I think the key is in classifying the person. I don’t think you can apply a cookie-cutter type system. Benefits would be getting better faster, getting back to work faster, education in self-management. At the end of the day it saves money for patients and the healthcare system". (ON5)* |
|  | I believe the disadvantages of not using SCA include slower recovery, lower patient satisfaction, less self-management and autonomy, longer treatment time, higher costs, poorer standard of care | *"Clinicians would be swimming in a lot of information. They wouldn’t feel that they have a coherent approach to follow or an end point that they’re going towards. It could prolong visits and the financial ramifications, for the patient, insurance companies, client could be off work longer, family, participation in life because they’re not getting better faster". (ON4)* |
|  | Outcomes I expect to see are less pain, better function, faster recovery, adherence to protocols, self-management, higher satisfaction, faster return to work, fewer visits, less medication | *"Usually I would expect a person to become more empowered, to be able to deal with their own pain and more able to deal with recurrences. Increased function, lower pain, increased ROM, decreased score on RMQ, increased score on photo. With the Mack model, a big thing that I find is an important outcome is the patient’s decreased fear of re-injury and a feeling that they can to an extent self-manage". (QC2)* |
| *Belief about capabilities* | I am confident in assessing NSLBP patients using SCA & determining the targeted treatments. | *"I’m very confident. For me it’s easy because I’m trained in a CS and I have certification but it can be difficult for someone who’s not trained". (ON6)* |
| *Behavioural Regulation* | I monitor changes in patients’ health status. | *"Yes I’ll see in terms of their ROM what that is like, if they’ve returned to work, how their pain levels are, and they’ll fill out another questionnaire". (ON3)* |
|  | I do not monitor changes in patient's health status. | *"No because I never see them again, I only assess them. I don’t do any treatment". (NS1)* |
|  | It would be helpful if there was more movement testing. | *"I will say that it is more difficult to use Mackenzie in a more elderly population if there is dementia because subjective questioning is hard and if they have difficulty identifying whether it is better or worse, but at least we can go by ROM or movement testing". (ON1)* |
|  | It would be helpful to have a strong subjective exam and core stabilization exercises. | *"The Mack method is really reliant on a strong and good subjective exam and even more. Also there are a lot of subgroups that would respond to core exercise, that’s not directly taught very much in Mack (core stabilisation exercises for example, Stewart McGill out East"). (AB3)* |
|  | It would be helpful to have team work for complicated cases. | *"I do find it helpful to treat alongside with other therapists. There is one therapist that I’ve worked with and we treat patients together and that is very helpful, those would be the more complicated cases. The different levels of learning are all useful". (AB4)* |
|  | It would be helpful for managers, clinicians and patients to be more aware about SCA. | *"I don’t know. Maybe more awareness from the medical side and from the patient side about these systems and how they are effective". (ON6)* |
|  | I have a clear plan under what circumstances I will use SCA in my practice. | *"Yes I have a clear plan. The assessment we’ve learned helps us to systematically classify people into the three categories". (ON5)* |
| *Skills* | I have been trained to use SCA. | *"I’ve done course on MIS and on the MDT approach. I’ve done all my A-D level on Mackenzie". (QC2)* |
|  | I have the necessary skills to use SCA. | *"Yes. Training, communication skills, the ability to properly take subjective and objective history and exam. A lot is being able to subcategorize your patients based on the actual assessment findings. It’s wouldn’t necessarily have to be Mackenzie. Patience and having open eyes and an open mind". (AB3)* |
|  | Skills required to treat patients with high risk of disability are: ability to screen, good communication, psychosocial training, teamwork. | *"I think part of it is definitely…so the clinician should be able to educate the patient on the importance of return to work, understanding more the psychosocial part of disability and how to treat that, I think the management of psychosocial comorbidities that occur with low back pain I guess". (ON6)* |
|  | Communication skills are extremely important for the management of LBP patients using SCA. | *It’s crucial yes very, very important. It’s probably one of the most important skills to have for LBP... I think communication is huge. I tie that in with education...". (NS1)* |
| *Intention* | I will manage all of the next 10 patients using SCA. | *"All of them". (ON7)* |
|  | I will manage most of the next 10 patients using SCA. | *"On average the number of patients that would be appropriate… and in about 7 out of 10 I follow through with it". (QC1)* |
| *Goals* | The goal of managing NSLBP patients with SCA is not incompatible with achieving another objective. | *"I wouldn’t say that I’ve ever noticed that using Mackenzie has limited me in achieving any other goals". (ON7)* |
|  | The goal of managing NSLBP patients with SCA can be incompatible with achieving another objective. | *"It depends on the clinic. One of my colleagues had trouble with the clinic owners because people were getting better too quickly and it was bad for business. It could be incompatible if someone had a goal or you felt you had a need to increase their core strength… With psychological overlay patients...You may have to hold off of using Mackenzie until you addressed the major fear of movement". (AB3)* |
| *Memory, Attention & Decision* | Deciding if a patient should be managed using SCA is easy. | *"Very easy". (ON1)* |
|  | Deciding if a patient should be managed using SCA is not easy. | *"There are some challenging ones. Within 3-5 visits is the guideline I use". (AB1)* |
|  | The rule of thumb I use is the SCA. | *"I don’t use any rules I only use Mackenzie". (AB1)* |
|  | The rule of thumb I use is research and effectiveness. | *"Consult the research. I try to see things in terms of probability of applicability. In using which method would I have the highest proportion of responders if I tried to use that method. And if that is not effective then I would move on the next type of approach". (QC1)* |
|  | The rule of thumb I use is the mechanical component in the history. | *"If there’s a clear mechanical component in the history to the patient presentation then I will start with that. If not, then this is when I have to ask if I should be assessing this patient or not. I go based on the history and if I find something it’s easy for me to have a baseline in the clinic and verify the mechanical behaviour of the pain then I will use that SCA". (QC2)* |
|  | The rule of thumb I use is patient compliance. | *"You can get an idea based on demographics and a conversation, you might be able to figure out how compliant this person may be to your treatment plan". (ON6)* |
| *Reinforcement* | I would manage NSLBP most of the time using the SCA because rewards are greater and patients are satisfied. | *"I’d say 5. I use it on every client regardless". (ON4)* |
| *Environmental Context and Resources* | Barriers to using SCA include lack of time, money, environmental constraints, other colleagues, lack of expertise, patient preference, language, unmotivated patients. | *"Yes, scheduling for sure, having enough time for individual patients, also the cohesiveness and the culture of the clinic, so other practitioners are on the same page, this is a huge thing. So I was recently away for 10 days and I had no problem booking my patients with other therapists because I knew there’s been consistency of care and of approach. Also having a variety of skills in the clinics, I’ve got access to other clinicians in the same clinic that can take a look at the patient for a few visits if I’m getting stuck. It’s very much a team environment". (AB1)* |
|  | There are no barriers to using SCA. | *"I don’t think so. Nothing I can think of. I always apply it even if I have limited time. If the clinic owner had other objectives in mind, I’ll still do it anyway". (ON3)* |
|  | Facilitators to using SCA include: need for fewer sessions, having private room, autonomy, team work, support from management. | *“The support from senior management, a CEO level encouraging us to take more courses specifically in relation to taking more MDT courses so we can improve our use of it as a SCA. If we had funding or reimbursement to be able to learn more, that would be improved.” (ON7)* |
|  | No onsite rehabilitation equipment is required. | *"The system I uses doesn’t require any special equipment". (ON4)* |
|  | Some onsite equipment is required. | *"There is some equipment needed. That is why this system is used in a lot of places because you can screen patients with Skype, I know it’s used with the armed forces when they don’t access to a lot of equipment but they still find it to be very effective. I use a plinth, a stool, simple things like therabands, balance equipment but when the system’s working well, there’s no need for electrical modalities like TENS or ultrasound. We could probably get rid of a lot of the passive care type treatment tools". (AB1)* |
|  | There are resources available that help me manage patients using the SCA. | *"There is a page in the Journal of Sports Physiotherapy. A one page educational tool. I do have Treat your own Back, Active Lumbar Rolls and I use videos". (ON5)* |
| *Social Influences* | I would consider consulting more experienced practitioners if I need help. | *"Yes. If the pieces of the puzzle are not fitting together I will sometimes ask a colleague to do an assessment". (AB2)* |
|  | I would not consider consulting my colleagues about SCA. | *"It’s something we have debates about". (AB1)* |
|  | The views of other colleagues do not influence my decision to manage patients using SCA. However, The views of researchers influence my decision to manage patients using SCA. | *"I would be mostly influenced by what the research says". (QC1)* |
|  | The views of other colleagues influence my decision to manage patients using SCAs. | *"Strongly when I’ve talked to other practitioners that use the system and see the results, that’s what led me to use it". (ON6)* |
|  | Having an acute patient in apparent distress would not influence my decision to manage such patients using the SCA. | *"Not at all. It will push me further to look at them with it". (ON1)* |
|  | I will try to deal with psychosocial variables by addressing stress using psychosocial screening. | *"I will then refer back mostly to my training in dealing with psychosocial variables. Explain Pain by David Butler. First address the stress first by doing psychosocial screening". (QC1)* |
|  | Having a chronic patient with important psychological overlay would not influence my decision to manage with SCA. | *"I’m still going to use itز But I’ll still use that classification system and that won’t hinder my decision". (ON3)* |
|  | Having a chronic patient with important psychological overlay would influence my decision to manage with SCA. | *"Less likely. Because I feel like it’s probably less effective". (ON6)* |
|  | With some psychosocial issues, I feel I’m not 100%. | *"With some psychosocial issues, I feel I’m not 100%". (ON5)* |
| *Optimism* | I am generally optimistic regarding the added value of using SCA, in my daily practice. | *"Very optimistic yes". (ON3)* |
|  | I am not sure if I am optimistic about the added value of using SCA in my daily practice. | *"Optimistic? I don’t know. I think I’m confident that I’m giving the best I can to my patients. Does it make me more optimistic? Hm. If I can give them what they need to do, or if I say no, you need to see a rheumatologist, I think that’s important to know for the patient. I think it’s more confidence than optimism. I know I can rely on it. I know that". (ON2)* |
| *Social Professional identity* | I consider using SCA to be part of my work as a physiotherapist. | *"Yes definitely it plays a big role in me as a physiotherapist. Because it’s not just for LBP, It’s for any body part". (QC1)* |
|  | I think it is appropriate that my role should include managing patients with non-specific LBP using the SCA. | *"Yes it is. The same reason, it’s appropriate because it’s what’s right". (AB2)* |
