# Supplementary material for: Professional barriers and facilitators to using stratified care approaches for managing non-specific low back pain: a qualitative study with Canadian physiotherapists and chiropractors
Source: Chiropr Man Therap. 2019 Dec 13;27:68. doi: 10.1186/s12998-019-0286-3 (PMC6909494; doi:10.1186/s12998-019-0286-3)
Supplement: Supplementary file 3 — Additional file 3. Findings of relevant domains together with illustrative quotes for chiropractors. [file 12998_2019_286_MOESM3_ESM.docx]

**Additional file 3: Findings of relevant domains together with illustrative quotes for chiropractors**

| **TDF Domains** | **Specific beliefs** | **Selected statements** |
| --- | --- | --- |
| *Knowledge* | I am aware of existing SCA. | *"Yes. I’m aware of Mack, the SB and the Core Back Tool". (QC1)* |
|  | My understanding about the use of SCA is to classify patients into groups to provide effective treatment for each group. | *"Basically it identified patterns or symptomatology and then identifying particular approaches that could be followed up for care with patients. I think it would be very similar throughout the different systems". (ON2)* |
|  | SCAs can streamline different practitioners' work. | *"As far as effectiveness for it, to date, I don’t know of anything that’s more effective globally but there could be other systems that are more effective". (ON1)* |
|  | There are mechanistic and prognostic SCAs. | *"There are different stratification systems. Some are mechanistic, some are predictive….". (ON7)* |
|  | I have limited understanding how to use SCAs. | *"I don’t know a lot about them. We go for more individualized programs based on functional assessment". (AB2)* |
|  | I agree with the recommended use of SCA for LBP patients. | *"I would say I would agree". (ON2)* |
|  | I do not necessarily agree with the recommended use of SCA for LBP patients. | *"Not necessarily (agree)". (MB1)* |
|  | I know how to use SCA to target the management of non-specific LBP patients. | *"Yes I know how to use it". (QC2)* |
|  | I know how to use SCA to target the management of non-specific LBP patients but I don't necessarily use it or I use it alongside other things. | *"The only one that I would be very familiar with would be the CBT although I don’t necessarily incorporate it into my practice, although it’s the way I practice anyway". (ON2)* |
|  | I do not know how to use SCA to target the management of non-specific LBP patients. | *"I know like the Start Back has a way you can monitor that but I haven’t used that yet". (AB1)* |
| *Belief about consequences* | I believe the advantages of using SCA include better managing patients and evidence-based practice. | *"Since we’ve introduced stratification we are getting to patient goals quicker, we have increased patient satisfaction, it’s even better now. We’re getting a lower VAS scores sooner and lower disability scores sooner, and quicker return to ADLs and return to work". (ON7)* |
|  | I believe the disadvantages of not using SCA include poor management of patients and not evidence-based practice. | *"I guess inappropriate interventions would be one. I don’t think it looks great on the profession when they’ll go to 5 different chiropractors or physiotherapists and get 5 different diagnoses". (ON3)* |
|  | I believe the disadvantages of using SCA include wrong management of the patients, more focus on yellow flags, require memorizing, time consuming, cultural differences. | *"I’ve got no time to get them to fill in a form. I’ve got 10 minutes set aside for them. I don’t have half hour or 40 minutes for them. I’ve got to do a very rapid examination. They never book and say they need a complete examination. They just show up on a regular routine visit". (MB1)* |
|  | Outcomes I expect to see are less pain, better function, faster recovery, adherence to protocols, self-management, higher satisfaction, faster return to work, fewer visits, less medication. | *"I’d hope to see increased function, decreased disability and decreased pain". (ON2)* |
| *Belief about capabilities* | I am confident in assessing NSLBP patients using SCA & determining the targeted treatments. | *"I am very confident". (QC2)* |
|  | I am not confident in assessing NSLBP patients using SCA & determining the targeted treatments. | *"Not confident at this current moment...". (ON2)* |
|  | Decisions based on my experience are more important than using SCAs. | *"I’m just not convinced that 7 questions are better than my personal experience really". (MB1)* |
| *Behavioural Regulation* | I monitor changes in patients’ health status. | *"After the initial assessment I do a series of treatment and I do a reassessment. This is the monitoring. Positive Physical Assessment test and also questionnaire, Oswestry and reassessment questionnaire". (QC1)* |
|  | I do not use a specific method to monitor changes in patients’ health status. | *"I don’t use any specific method necessarily. In a general sense a lot of patients I see for an acute episode if they’re resolved I may not see them for a while. A standard re-introduction I don’t use anything more specific than that. General health changes". (ON4)* |
|  | It could help if SCAs were more available. It could help to be better able to interpret the results, impact on prognosis and indirect cost. | *"In terms of outcome measures that I could use as I proceed, in terms of treatment: Using more readily available outcome measures to re-evaluate patients and know the importance of what I see when I measure those. If I get a change in 30% improvement in kinesophobia scale after x amount of treatment how does that impact in the long term. Am I changing the outcome? To have that kind of answer would be very useful. What I haven’t seen how much to stratify helps to address which psychosocial factors are present to get a firm estimate of that patients chance of responding to treatment. And how much improvement I can expect long-term for this patient. To get answers to how does it affect the rate of recurrent, how does this affect the long-term use of care, or investigations, indirect costs". (QC2)* |
|  | It would be helpful to have SCAs specifically designed for chiropractic. | *"Probably if it was customized a little bit more. The one complaint I have about the Start Back in particular is that it’s very physio centred. It’s very centred towards physios and general practitioners. Chiropractors we see in Canada at least a very high percentage of back pain patients. Those would probably be the big things". (AB1)* |
|  | It could be helpful to use tools for clinicians to monitor pain and disability. | *"Yes as a clinician to facilitate implementing things, a toolkit of some manner helps. So for example, the VAS score which is well established, you have a way of monitoring, the Oswestry or Roland Morris you take a baseline, at mid-treatment and you have comparison over time. If there’s an intention to facilitate utilizing SCA and implementing it and monitoring changes then there should be an effort to make easy to use tools for clinicians to monitor those parameters that you want to observe". (ON7)* |
|  | It could be helpful to use one SCA that is adopted and widespread. | *"More consistency would be helpful. That would help me adopt an approach. In the utilization something like simple reminders, or training programmes, or at my fingertips reminders to utilize a particular approach". (ON2)* |
|  | It could be helpful to have computerized records to ease tracking. | *"I think if I went to computerized records that would be easier to track". (MB1)* |
|  | I have a clear plan under what circumstances I will use SCA in my practice. | *"The SCA are typically used with any new patient who comes in with LBP. Motivation would be dependent on how the interview is performed. So if the patient is resistant in the interview doesn’t allow us to capture a full patient history. Motivational interview skills". (ON8)* |
|  | I don't have a clear plan under what circumstances I will use SCA in my practice and having one would not help. | *"I don’t have a clear plan. I continue to motivate them. I try to educate them.  I don’t think having a plan would help me to motivate them. Self-management and active care I utilize with all my patients. I do educate them as to what the evidence states. I don’t that would make a difference from the patient perspective". (ON2)* |
| *Skills* | I have been trained to use SCA. | *"Yes. I’m trained in Core and Keele". (ON6)* |
|  | I have not been trained to use SCA. | *"No. Self-trained, just reading online and using online resources". (ON7)* |
|  | I feel that I have the necessary skills to use SCA. | *"Yes". (AB2)* |
|  | Skills required to treat patients with high risk of disability are: ability to screen, good communication, psychosocial training, teamwork, and strong training. | *"I think a strong background in critical appraisal and to be familiar with the literature in this domain so you can identify those patients in the first place, inter-professional skills both verbal and written so you can communicate with other medical practitioners to make sure referrals are made appropriately. Understanding of the theoretical frameworks so you understand .... and understand and formulate a prognosis is very important, and the ability to communicate with your patients...". (ON5)* |
|  | I am not sure what skills are required to manage patients at high risk of disability using SCA. | *"I don’t know if there’s anything a classification system can give me in terms of higher risk cases. I feel that it’s relatively black and white. Either the patient is going to response by putting them into one of these classification system and doing the movements and exercises with and for them or they’re not....". (ON1)* |
|  | Learning SCAs don’t require courses. | *"Because otherwise you have to take a ton of courses. So there has to be an easier way than that. SCAs don’t require you to go to all those courses. You can figure them out from the comfort of your own home". (AB1)* |
|  | Communication skills are extremely important for the management of LBP patients using SCA. | *"I think it’s massive, it’s highly important to be able to communicate with patients, other care providers, your colleagues, if you can’t communicate what’s going on and what’s you’re concerned about in the management of the patient, you could really do them a disservice and even harm them". (ON6)* |
| *Intention* | I will manage all of the next 10 patients using SCA. | *"I would say 10". (ON8)* |
|  | I will not manage any my patients using SCA. | *"None (sorry!). I absolutely could and my approach is very similar but it’s not currently part of my practice". (ON2)* |
|  | I would manage only who needs SCA. | *"I don’t know. That depends on what they need. Out of 10 on average I would say 1-2 out of 10." (ON4)* |
| *Goals* | The goal of managing NSLBP patients with SCA is not incompatible with achieving another objective. | *"No, I don’t think it would interfere". (AB1)* |
| *Memory, Attention & Decision* | Deciding if a patient should be managed using SCA is easy. | *"Piece of cake". (ON7)* |
|  | The rule of thumb I use is the clinical presentation of the patient. | *"We base it on the diagnosis. We base it on the patient’s goals. We base it on the more advanced MSK examination, on the patient’s capacity and functional limitations". (AB2)* |
|  | I do not use a rule of thumb. | *"I wouldn’t say I have any rules". (ON1)* |
|  | The rule of thumb I use is the guidelines. | *"I guess the guidelines and knowing that really always they should be used. As a rule of thumb, it’s always good to use". (ON5)* |
|  | The rule of thumb I use is based on the SCA's simplicity. | *"Simplicity of use". (ON8)* |
|  | I am not sure which SCA to apply with my patients. | *"We don’t use them all concurrently, all the time and I don’t know how I’d include them all at the same time. Because they’re different. To incorporate SB at the same time as Core I’m not doing that". (ON7)* |
| *Reinforcement* | I would manage NSLBP most of the time using the SCA because rewards are greater and patients are satisfied. | *"5 I suppose. All the time. That’s what we try to do, to have the best possible patient outcomes". (ON3)* |
| *Environmental Context and Resources* | Barriers to using SCA include lack of time, lack of training, environmental constraints, seeing fewer patients, cost. | *"Other than the more… time factor, just the medico-administrative, how they fill out the questionnaire is the main obstacle which I am working to correct". (QC2)* |
|  | Facilitators to using SCA include: having certified colleague in the team, having easy-to-use questionnaire-based SCAs. | *“Time constraints aren’t really an issue for us. We have an hour per appointment and 30 minutes for follow up. Our physiotherapist is certified Mack (full diploma) so his paperwork is also the Mack paperwork. It’s a multidisciplinary clinic.” (ON3)* |
|  | There are no major barriers to using SCA. | *"No I don’t think so". (ON4)* |
|  | No onsite rehabilitation is required. | *"No I don’t think that there’s specialized equipment necessary. I don’t see that as a barrier". (ON2)* |
|  | Onsite rehabilitation may be required | *"Absolutely. Why? Ease of access. This equipment would be essential. I believe so". (AB2)* |
|  | There are resources available that help me manage patients using the SCA. | *"I use CCA Straighten Up App and I give them handouts. I have information on the website". (MB1)* |
|  | There are no resources available to help me manage patients using the SCA. | *"No not really. Like I said most of them are fairly desktop decisions. So there doesn’t seem to be anything that would either hinder or enhance my ability to use them that I can think of. " (AB1)* |
| *Social Influences* | I would not consider consulting more experienced practitioners. | *"I don’t think so." (ON2)* |
|  | I would consider consulting other staff but not chiropractic colleagues. | *"Fellow staff members at my own office I do that quite often actually. Other colleagues probably not". (ON4)* |
|  | I would consider consulting more experienced practitioners if I need help. | *"I would definitely speak to other colleagues but not on the need to use SCA for NSLBP". (ON6)* |
|  | The views of other researchers influence my decision to manage patients using SCAs. | *"Yes. In the context of research and education it influences my position strongly." (QC2)* |
|  | The views of other researchers do not influence my decision to manage patients using SCAs. | *"I’m not really influenced by others. So not at all". (ON1)* |
|  | The views of other researchers may or may not influence my decision to manage patients using SCAs. | *"I think that it’s affected me both positively and negatively. I definitely was a victim of it after doing the workshop and after speaking to a number of my colleagues at the chiropractic college that I highly respect, and I was very excited about the idea of implementing it and introduce it to the student body, and I was 100% gung-ho on it and then when I heard the backlash of other colleagues and practitioners that I really respected and how they were hesitant to implement that strategy, that confused me and I thought maybe it’s not as amazing as I originally thought. But generally speaking I have been very positively swayed by the response of most of my colleagues from the college. Inside my practice they’ve all been negative about it. ". (ON6)* |
|  | Having an acute patient in apparent distress would influence my decision to manage such patients using the SCA. | *"Yes I mean. So psychological distress is a psychosocial risk factor so it is likely going to affect my stratification of that patient. It will increase my level of risk of chronicity". (QC2)* |
|  | Having an acute patient in apparent distress would NOT influence my decision to manage such patients using the SCA. | *"No I implement the recommendations of the stratification system but without actually diagnosing them within the classification". (ON6)* |
|  | Having an acute patient in apparent distress might or might not influence my decision to manage such patients using the SCA. | *"It would depend if I feel that their acute presentation was more of an emotional response or fear avoidance or am I seeing hard physical signs of distress? But as far as SCA if I feel there’s a lot of malingering signs or magnification, then they’ve got to be handled very much differently". (MB1)* |
|  | Having a chronic patient with important psychological overlay would NOT influence my decision to manage with SCA. | *"No again I think it really reinforces the importance of SCA. I would be more concerned with using SB and more concerned with collaborating with other HCP if there was that significant psychological overlay but my approach would remain the same". (ON5)* |
|  | Having a chronic patient with important psychological overlay would influence my decision to manage with SCA. | *"Likely yes. I suppose through clinical care and regards to my own experience, I’m not sure that a specific SC model I know of encompasses the majority of chronic pain patients". (ON3)* |
| *Optimism* | I am generally optimistic regarding the added value of using SCA in my daily practice. | *"I would say I’m generally optimistic about the value of them but I have yet to incorporate them into my daily practice". (ON2)* |
| *Social Professional identity* | I consider using SCA to be part of my work as a chiropractor. | *"Yes. Because as a clinician I believe it’s important for us to be proficient but also evidence-based". (ON8)* |
|  | I do not consider using SCA to be part of my work as a chiropractor. | *"No, currently no I guess I don’t see it as a necessary part of my work currently..." (ON2)* |
|  | I think it is appropriate that my role should include managing patients with non-specific LBP using the SCA. | *"Yes absolutely. What else are we supposed to do? To me, it’s just what should happen". (ON4)* |
